# Genetic Ancestry, Intrinsic Tumor Subtypes, and Breast Cancer Survival in Latin American Women

**DOI:** 10.1158/2767-9764.CRC-25-0014

**Published:** 2025-07-03

**Authors:** Daniela Alves da Quinta, Darío Rocha, Cristian Yáñez, Renata Binato, Sheila Coelho Soares-Lima, Xiaosong Huang, Daiana Ganiewich, Valentina A. Zavala, Monica Sans, Alejandra Lopez-Vazquez, Jael Quintero, Olivia Valenzuela, Antonio Quintero-Ramos, Alicia Del Toro-Arreola, Mauricio Cerda, Katherine Marcelain, Susanne Crocamo, Maria Aparecida Nagai, Dirce M. Carraro, Marcia Maria Chiquitelli Marques, Jorge Gómez, Nora Artagaveytia, Adrian Daneri-Navarro, Bettina G. Müller, Javier Retamales, Carlos Velazquez, Elmer A. Fernández, Osvaldo L. Podhajcer, Eliana Abdelhay, Ricardo A. Verdugo, the LACRN Investigators, Andrea S. Llera, Laura Fejerman

**Affiliations:** 1 Laboratorio de Terapia Molecular y Celular, Fundación Instituto Leloir-CONICET, Ciudad de Buenos Aires, Argentina.; 2 Universidad Argentina de la Empresa (UADE), Instituto de Tecnología (INTEC), Buenos Aires, Argentina.; 3 Universidad Nacional de Córdoba, Facultad de Ciencias Exactas, Físicas y Naturales, Córdoba, Argentina.; 4 Programa de Genética Humana, ICBM, Facultad de Medicina, Universidad de Chile, Santiago, Chile.; 5 Instituto Nacional de Câncer, Rio de Janeiro, Brazil.; 6 Genome Center, College of Biological Sciences, University of California Davis, Davis, California.; 7 Department of Public Health Sciences, University of California, Davis, Davis, California.; 8 Hospital de Clinicas Manuel Quintela, Universidad de la República, Montevideo, Uruguay.; 9 Universidad de Sonora, Hermosillo, Mexico.; 10 Universidad de Guadalajara, Guadalajara, Mexico.; 11 Integrative Biology Program, Instituto de Ciencias Biomedicas (ICBM), Centro de Informática Mádica y Telemedicina, Facultad de Medicina, Universidad de Chile, Santiago, Chile.; 12 Departamento de Oncología Básico Clínico and Centro para la Prevención y el Control del Cáncer (CeCAN), Facultad de Medicina, Universidad de Chile, Santiago, Chile.; 13 Instituto de Cancer de São Paulo, São Paulo, Brazil.; 14 AC Camargo Cancer Center, São Paulo, Brazil.; 15 Hospital de Cancer de Barretos, Barretos, Brazil.; 16 Health Sciences Center, Texas A&M University, Bryan, Texas.; 17 Instituto Nacional del Cáncer, Santiago de Chile, Chile.; 18 Grupo Oncológico Cooperativo Chileno de Investigación, Santiago de Chile, Chile.; 19 Fundación para el Progreso de la Medicina, ScireLab, Córdoba, Argentina.; 20 CONICET, Córdoba, Argentina.; 21 FCEFyN, Depto. de Computación, Escuela de Ingeniería Biomédica, Universidad Nacional de Córdoba, Córdoba, Argentina.; 22 The list of LACRN Investigators with their affiliations is included at the end of the Supplementary Information.; 23 Department of Public Health Sciences, School of Medicine, University of California Davis, Davis, California.; 24 University of California Davis Comprehensive Cancer Center, School of Medicine, University of California Davis, Davis, California.

## Abstract

This study investigates the relationship between genetic ancestry, breast cancer subtypes, and survival outcomes among 951 locally advanced breast cancer cases from Argentina, Brazil, Chile, Mexico, and Uruguay, participating in the Molecular Profile of Breast Cancer Study. Array-based genotyping and ADMIXTURE analysis were used for genetic ancestry evaluation. Breast cancer subtypes were defined by IHC and the gene expression–based PAM50 algorithm. The distribution of genetic ancestry, including European, Indigenous American (IA), African (AFR), and East Asian components, revealed a heterogeneous genetic admixture across countries, with the highest IA ancestry observed in Chile (30.9%) and Mexico (30.8%). Testing the relationship between genetic ancestry and breast cancer subtypes demonstrated that a 10% increase in European ancestry was significantly associated with a 14% decrease in the odds of developing HER2-enriched breast cancer, after adjustment by age, nodal status, and the AFR component (adj. *P* = 0.021, luminal A as reference). Accordingly, a 10% increase in IA ancestry was associated with a 21% increase in the probability of having HER2-enriched breast cancer (adj. *P* = 0.022). IA ancestry also significantly increased overall survival after adjustment by age, nodal status, and AFR ancestry, although this result is controversial and may be affected by the size and heterogeneity of the Molecular Profile Breast Cancer Study cohort. Our research confirms previous findings of a high prevalence of HER2-dependent breast tumors among Hispanic/Latina women and strengthens the hypotheses of the existence of either population-specific genetic variant(s) or of other ancestry-correlated factors that impact HER2 expression in breast cancer consistently across different Latin American regions.

**Significance:**

The evidence in this work supports the idea that factors linked to genetic ancestry influence the prevalence of breast cancer subtypes in Latin America, potentially affecting treatment needs in the region.

## Introduction

Breast cancer is the leading cause of cancer death in Latin American women (1). In this region, breast tumors are frequently diagnosed at stages II or higher, mainly because of relatively low rates of mammography screening (2, 3) and delays in access to care for diagnostic studies (4). Breast cancer is also a heterogeneous disease including tumors with different responses to treatment and survival, and more aggressive subtypes are often associated with more advanced stages at diagnosis (5). Although some studies have shown a higher proportion of aggressive breast cancer subtypes in the region (6), in general, Latin American studies are not population-based and tend to overrepresent aggressive disease. However, registry-based studies in the United States have reported higher proportions of hormone receptor–negative (HR−) disease and HER2-positive (HER2+) disease in Hispanic/Latina women, suggesting that observations in Latin America might reflect a real difference in subtype distribution (7). An analysis conducted on patients with breast cancer from Peru showed an association between Indigenous American (IA) ancestry and HER2+ disease, which was replicated in an independent study including Colombian and Mexican women (8). Genetic ancestry is correlated with both genetic and nongenetic factors (9, 10), therefore, additional testing of the observed association, including better characterization of tumor heterogeneity in women from multiple regions, could give important insights leading to the refinement of our understanding of relevant factors.

IHC markers (HR and HER2) are routinely tested in Latin American patients; however, data defining intrinsic molecular subtypes have been sparse. The Molecular Profile of Breast Cancer Study (MPBCS) of the Latin America Cancer Research Network (LACRN) collected molecular, clinical, and epidemiologic data from an observational cohort of more than 1,200 Latin American patients with breast cancer. Previous publications of the MPBCS have described the distribution of PAM50 subtypes from transcriptomic data and their impact on survival (11, 12), showing a worse prognosis for basal-like tumors [followed by HER2-enriched (HER2E) and luminal B tumors] than for luminal A (LumA) cases, in concordance with other advanced breast cancer cohorts. To test whether the degree of genetic admixture presented in patients of this study might correlate with breast cancer subtype distribution and survival, we evaluated the association between genetic ancestry, tumor subtype, recurrence, and survival in a sample of 951 women from public-health institutions in Argentina, Brazil, Chile, Mexico, and Uruguay participating in the MPBCS. We particularly focused on the intrinsic subtypes as they recapitulate the actual driver pathways of breast cancer more closely and estimate survival more precisely than IHC-based classifications (13).

## Materials and Methods

### Study participants

The MPBCS detailed eligibility criteria have been described previously (11). Briefly, women with clinical stage II or III (American Joint Committee on Cancer 7) breast adenocarcinoma were deemed eligible for this study. Patients with bilateral or inflammatory breast cancer or metastatic disease were excluded. As recruitment occurred prior to the collection of pathologic diagnosis samples to facilitate the acquisition of additional tissue for molecular studies, eligibility was confirmed retrospectively. Patients who were subsequently reassessed as stage I remained eligible for participation. The protocol received approval from the NCI Ethics Committee and local institutional review boards in each country. The MPBCS was registered at ClinicalTrials.gov (identifier: NCT02326857) and adhered to the principles of the Declaration of Helsinki and local regulations. Before the study procedures, all participants signed the study-specific written informed consent form. Participants were monitored for 5 years to track survival and recurrence. Electronic case report forms were utilized to capture clinical data, with local data managers ensuring accuracy.

Tumor and blood samples were collected at the time of diagnosis, prior to the initiation of any treatment. From a total of 1,278 patients, DNAs extracted from blood samples from 1,001 eligible patients were successfully genotyped, and 951 constituted the final dataset. From those, 827 also had transcriptomic information available from treatment-naïve tumor samples and constituted the dataset used for association and survival analyses.

### Demographic data

After signing the consent form, trained study personnel applied a questionnaire containing questions about socioeconomic and demographic characteristics and lifestyle factors, including age, education, alcohol and tobacco use, access to healthcare, familial cancer, hormonal and reproductive history, and physical activity (14).

### Clinical data

Clinical stages for each patient were established according to the American Joint Committee on Cancer staging manual seventh edition. Given that some patients underwent chemotherapy before surgery, the lymph node status at diagnosis was defined clinically as negative (i.e., no clinical evidence of involved nodes) or positive (when at least one node was detected at physical examination). Body mass index was calculated using height and weight reported in medical charts. IHC for HR [i.e., estrogen receptor (ER) and progesterone receptor (PR)], HER2, and Ki67 were determined locally following standard operating procedures (11). All local pathology departments were accredited by the College of American Pathologists. Patients were classified according to IHC subtype based on ER/PR and HER2 status. We used a cutoff of 1% to define ER/PR positivity. HER2 positivity was defined 3+ staining by IHC or 2+ with positive gene amplification by FISH or chromogenic in situ hybridization testing. Detailed information regarding molecular subtype determination (PAM50 subtypes: LumA, luminal B, HER2E, and basal-like) by microarray-based transcriptomic assay has been described previously (11). Quality control measures, including principal component analysis, were implemented to avoid bias. Patients classified as normal by PAM50 were not considered in the analyses.

### Genetic ancestry estimation

DNA was extracted from the 1,001 available whole blood samples following standard protocols. DNA samples were genotyped with Infinium Multi-Ethnic Global-8 (MEGA) array version 1.0 (Illumina WG-316-1004), comprising 1,748,250 markers (single-nucleotide variants and insertions/deletions). Genotypes were obtained in GenoType Compressor format using the Illumina Array Analysis Platform Genotyping version 1.1.0 GenCall program. The genotypes and probes of each marker were aligned per sample against the hg19 reference genome with bcftools (https://samtools.github.io/bcftools/) and gtc2vcf (https://github.com/freeseek/gtc2vcf), obtaining a binary variant call format file (BCL). Quality control was performed using PLINK 1.9 (RRID: SSR_001757; ref. 15). All variants that were not present in at least 10% of the samples were removed. Variants were filtered by a Hardy–Weinberg equilibrium test, discarding 9,248 variants with a *P* value lower than 1E-7. Fifty samples with a genotyping call rate of less than 90% were eliminated. From the remaining samples, 391,714 monomorphic variants were eliminated using a minor frequency allele filter >0 (0%). After filtering by 1% minor frequency allele, 873,197 variants were available in the final set of 951 samples.

The following two genome datasets were used to generate the IA ancestry reference group: (i) 17 genomes of Patagonian origin (3 from Laitec Island of putative Chono ancestry, 4 Kaweskar, 3 Huilliche, 3 Pehuenche, and 4 Yamana) and (ii) 21 unrelated individuals of IA origin from Mexico, Brazil, Argentina, Peru, and Colombia [from the Simons Genome Diversity Project (16)], including 1 Chane, 3 Karitiana, 2 Surui, 2 Piapoco, 2 Mayan, 2 Mixe, 2 Mixtec, 2 Pima, 2 Zapotec, and 3 Quechua. All individuals used as IA reference showed >90% IA ancestry in a previous ADMIXTURE analysis (see below). As we had a limited number (*n* = 38) of IA reference genomes, we decided to match as closely as possible the number of reference individuals in the other ancestries to have a similar level of information for each ancestry. Thus, 40 representative samples from Southeast Asia (40 Han Chinese, Europe (20 Iberian and 20 Northern Europeans from Utah), and Africa (40 Yoruba) were randomly selected among the 1000 Genomes phase three individuals with >90% specific ancestry (17). All three sets were converted to PLINK format independently. Hardy–Weinberg equilibrium filtering discarded those variants with a *P* value lower than 1E-7 in the three sets. Triallelic variants were also removed.

A set of reference samples plus those from the MPBCS were created with the intersection of 165,820 variants. After filtering by linkage disequilibrium with PLINK 1.9 (window size 50, number of variants 5, and variance inflation factor threshold 1.2), 41,054 variants were available.

Genetic ancestry estimation, defined as the estimated genetic similarity to reference populations, was performed using the unsupervised ADMIXTURE version 1.23 program (RRID: SCR_001263; ref. 18) with four populations to capture IA, European (EUR), African (AFR), and East Asian (EAS) ancestry based on the known major continental influences to the population of Latin America. As a result, individual fractions of the EUR, IA, AFR, and EAS ancestral components were obtained for the 951 samples. To visualize the ancestral structure of the MPBCS participants we conducted principal component analysis using the program PLINK 1.9.

### Statistical analyses

To evaluate the significance of differences in the distribution of variables among countries, χ^2^ tests (stats, RRID: SCR_025968) were used for categorical variables with Cramer’s V [*rcompanion* R package (19)] as a measure of the strength of association. Age was tested as a continuous variable using a Kruskal–Wallis test (stats, RRID: SCR_025968). The univariate association between EUR ancestry and breast cancer subtype was tested using a Kruskal–Wallis test with a *post hoc* Dunn test and Benjamini–Hochberg *P* value correction.

Multinomial logistic regression models were applied to study the association of the scaled EUR ancestry (i.e., 1 unit difference in the ancestry coefficient is equivalent to a change in 10% of the ancestry component) with breast cancer subtypes [HER2 status (negative or positive), IHC-based subtypes, or PAM50 subtypes] using the *nnet* R package (20). HER2−, HR+ HER2−, and LumA patients were defined as reference groups for each model, respectively. For studying the association between ancestry and survival, Cox proportional hazard regression models with the *survival* R package were performed considering ancestry as a continuous scaled variable (1 U equivalent to a change in 10% of the ancestry component). To select the most important potential confounders in the logistic and Cox models, we analyzed previous evidence in the literature and our own univariate and collinearity analysis (see Extended Figs. E1, E2, and E3 in Supplementary Information for a detailed analysis). Clinical nodal and tumor statuses were strongly correlated with clinical stage (coefficients of 0.78 and 0.57, respectively, Extended Fig. E3 in Supplementary Information), as they are parameters used to calculate clinical stage. For this reason, we chose clinical lymph nodal status (i.e., negative vs. positive) as the simplest and more complete (i.e., more subjects had this variable with data) confounder representative of stage. We also selected age at diagnosis as a continuous variable (correlation coefficients of 0.11 with EUR ancestry and −0.09 with IA ancestry), and AFR ancestry (coefficients of −0.35 with EUR ancestry and −0.01 with IA ancestry) and country (coefficients of 0.21 with EUR ancestry and 0.48 with IA ancestry) were included as potential confounders (Extended Fig. E3 in Supplementary Information).

All analyses were performed in R version 4.2.2. *P* values ≤ 0.050 were considered significant.

### Data availability

The processed data and scripts used for this study are available at https://github.com/danielaalvesdq/LACRN-MPBCS. Raw genotyping array data generated in this study are currently protected by data policies of the LAGENO-BC and Confluence Consortia and will be available from the corresponding author in the future upon reasonable request.

## Results

The distribution of relevant clinical and epidemiologic data of this hospital-based cohort is summarized in Table 1 and reflects the previously published description of larger versions of the cohort (11, 14). In this dataset of 951 patients, heterogeneity between countries was evident for age at diagnosis, education level, genetic ancestry, lymph node status, IHC-based and PAM50 subtypes, and survival but not for body mass index (Table 1). According to the Cramer’s V, the magnitude of the association between variables and country was weak (Cramer’s V ≤ 0.20).

**Table 1:** By-country epidemiologic and clinical characteristics of the 951 patients of the LACRN-MPBCS included in this study

Parameter	Total	Country					
		Argentina	Brazil	Chile	Mexico	Uruguay	Univariate analysis^a^
Number of patients included in this study	951	207	207	140	321	76	
Demographic/anthropometric							
Age at diagnosis–mean (SD)	951	55.6 (12.0)	52.5 (11.5)	56.2 (12.7)	52.1 (12.2)	58.4 (12.3)	***P* < 0.001** ^b^
Years of education–*n* (%)							***P* = 0.016 V = 0.13**
Up to 8 years	357	88 (42.5)	90 (43.5)	45 (32.1)	101 (31.5)	33 (43.4)	
9 years or +	338	81 (39.1)	73 (35.3)	70 (50.0)	97 (30.2)	17 (22.4)	
Unknown/missing	256	38 (18.3)	44 (21.2)	25 (17.8)	123 (38.3)	26 (34.2)	
BMI–*n* (%)							*P* = 0.532 V = 0.06
<25.0 kg/m^2^	231	57 (27.5)	56 (27.0)	32 (22.8)	64 (19.9)	22 (28.9)	
25.0–29.99 kg/m^2^	294	65 (31.4)	72 (34.8)	49 (35.0)	94 (29.3)	14 (18.4)	
>30.0 kg/m^2^	294	62 (29.9)	71 (34.3)	52 (37.1)	87 (27.1)	22 (28.9)	
Unknown/missing	132	23 (11.1)	8 (3.86)	7 (5.00)	76 (23.7)	18 (23.7)	
Ancestry–median (IQR)							
EUR	951	72.2 (59.2–87.4)	79.9 (63.5–90.7)	65.0 (62.1–68.4)	58.5 (52.6–66.5)	83.9 (76.0–91.0)	***P* < 0.001** ^b^
IA	951	22.9 (9.7–33.6)	5.3 (2.9–8.4)	30.9 (28.4–33.1)	30.8 (24.8–36.6)	10.4 (6.2–17.0)	***P* < 0.001** ^b^
AFR	951	1.2 (0.001–3.3)	11.9 (2.9–25.2)	0.1 (0.001–1.5)	3.8 (2.3–5.6)	3.2 (0.7–6.3)	***P* < 0.001** ^b^
EAS	951	2.8 (1.1–4.0)	0.03 (0.001–0.9)	3.2 (2.3–4.1)	5.6 (4.2–6.9)	1.0 (0.001–2.4)	***P* < 0.001** ^b^
Clinical							
Clinical stage–*n* (%)							***P* < 0.001 V = 0.19**
Early (IIA–IIB)	347	92 (44.4)	89 (42.9)	41 (29.3)	84 (26.2)	41 (53.9)	
Locally advanced (IIIA–IIIB)	583	114 (55.1)	117 (56.5)	98 (70.0)	223 (69.5)	31 (40.8)	
Missing/other	21	1 (0.5)	1 (0.6)	1 (0.7)	14 (4.3)	4 (5.3)	
Lymph node status–*n* (%)							***P* < 0.001 V = 0.22**
Negative	406	115 (55.5)	103 (49.7)	44 (31.4)	102 (31.8)	42 (55.3)	
Positive	526	92 (44.4)	103 (49.7)	95 (67.8)	206 (64.2)	30 (39.5)	
Missing	19	—	1 (0.6)	1 (0.8)	13 (4.0)	4 (5.2)	
HER2 status–*n* (%)							***P* < 0.001 V = 0.12**
Negative	730	171 (82.6)	153 (73.9)	107 (76.4)	236 (73.5)	63 (82.9)	
Positive	189	36 (17.4)	52 (25.1)	23 (16.4)	69 (21.5)	9 (11.8)	
Missing/equivocal	32	—	2 (0.97)	10 (7.14)	16 (4.98)	4 (5.26)	
IHC subtype–*n* (%)							***P* < 0.001 V = 0.11**
HR(+) HER2(−)	568	134 (64.7)	122 (58.9)	85 (60.7)	175 (54.5)	52 (68.4)	
HR(+) HER2(+)	108	19 (9.2)	36 (17.4)	13 (9.3)	35 (10.9)	5 (6.6)	
HR(−) HER2(+)	81	17 (8.2)	16 (7.7)	10 (7.1)	34 (10.6)	4 (5.3)	
HR(−) HER2(−)	154	37 (17.9)	31 (15.0)	21 (15.0)	57 (17.8)	8 (10.5)	
Missing	40	—	2 (1.0)	11 (7.9)	20 (6.2)	7 (9.2)	
PAM50 subtype–*n* (%)							***P* = 0.007 V = 0.10**
LumA	376	104 (50.2)	76 (36.7)	54 (38.6)	101 (31.5)	41 (53.9)	
LumB	189	32 (15.5)	44 (21.3)	37 (26.4)	55 (17.1)	21 (27.6)	
HER2E	112	20 (9.7)	24 (11.6)	17 (12.1)	44 (13.7)	7 (9.2)	
Basal-like	150	35 (16.9)	28 (13.5)	23 (16.4)	59 (18.4)	5 (6.7)	
Normal	46	16 (7.7)	6 (2.9)	6 (4.3)	16 (5.0)	2 (2.6)	
Missing	78	—	29 (14.0)	3 (2.3)	46 (14.3)	—	
5-year survival–*n* (%)							***P* = 0.015 V = 0.10**
Alive	748	166 (80.2)	157 (75.8)	112 (80.0)	254 (79.1)	59 (77.6)	
Dead	152	33 (15.9)	42 (20.3)	26 (18.6)	39 (12.1)	12 (15.8)	
Unknown/missing	51	8 (3.9)	8 (3.9)	2 (1.4)	28 (8.8)	5 (6.6)	

Abbreviations: AFR, African; BMI, body mass index; EAS, East Asian; EUR, European; HER2E, HER2-enriched; IA, Indigenous American; IHC, immunohistochemical; IQR, inter-quartile range; LumA, luminal A; LumB, luminal B.

Percentages (%) are defined as the proportion of individuals of a country that showed the variable level respect to the total individuals of such country (columns).

a χ^2^*P* value and Cramer’s V for categorical variables. Bold numbers highlight significant associations between the variable distribution and the country. A Cramer’s V value of 0.20 or less indicates a weak association, between 0.20 and 0.30 a moderate association, and higher than 0.30 a strong association.

b Kruskal–Wallis test for non-normally distributed continuous variables.

### Genetic ancestry distribution in the LACRN-MPBCS

**Figure 1:**
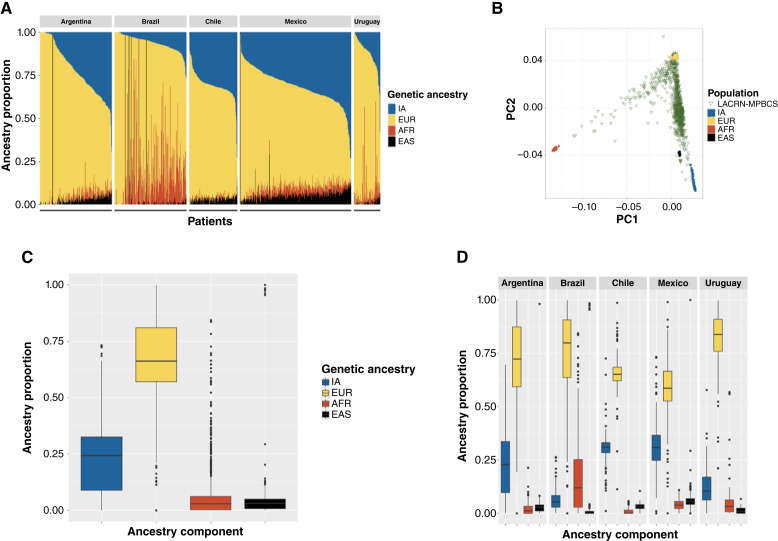
Population structure of the 951 patients with breast cancer of the LACRN-MPBCS cohort included in this study. **A,** ADMIXTURE ancestry estimations obtained assuming four ancestral components for each country of origin (**B**) Principal component analysis (PCA) of data from all LACRN-MPBCS patients (triangles) and 158 reference subjects (dots). The two principal components of variation are shown as PC1 and PC2. **C** and **D,** Boxplots showing the distribution of each ancestry component for the whole cohort and by country, respectively. Median ancestry and IQRs are depicted; whiskers extend up to 1.5 IQR. Values outside the inter-quartile range are shown as solid dots. AFR, African; EAS, East Asian; EUR, European; IA, Indigenous American.

The distribution of the ancestry components of this multicountry cohort showed important differences between individuals and countries (Fig. 1A–D). Study sites in Argentina, Uruguay, and Brazil showed the highest medians for EUR ancestry (72.2%, 83.9%, and 79.9%, respectively), whereas those in Chile and Mexico have the lowest EUR medians (65.0% and 58.5%, respectively). The AFR component is well-represented in Brazil (a median of 11.9%) and to a lesser extent in Mexico (3.8%) and Uruguay (3.2%), whereas it is minimal in Argentina and Chile. The IA median proportion was lowest (5.3%) in Brazil and highest in Chile and Mexico (30.9% and 30.8%, respectively; Table 1; Fig. 1D). A complementarity between the EUR and IA ancestries was evident for most individuals of the cohort (Fig. 1A), with the exception of Brazil in which the AFR component was most relevant (i.e., higher than 15% of the ancestry) in 40% (83/207) of patients. The EAS component was relatively low; only seven individuals were with more than 95% EAS ancestry, corresponding to patients identified as members of the Asian immigrant communities within each country.

At the whole-cohort level, the EUR (median of 66.1%) and IA (median of 24.2%) components were the most represented, followed by AFR (2.9%) and EAS (3.1%; Fig. 1A and C). When differences in the EUR median proportion among countries were tested, all intercountry comparisons were statistically significantly different (*P* < 0.050) except for Argentina versus Brazil and Uruguay versus Brazil, in which no differences in the representation of EUR ancestry were observed (*P* = 0.701 and *P* = 1.000, respectively). We also identified a difference in the EUR and IA ancestry variances between countries (Table 1; Fig. 1D). IQRs for Argentina (59.2–87.4), Brazil (63.5–90.7), and Uruguay (76.0–91.0) showed a larger variance in EUR coefficients than that of Chile (IQR = 62.1–68.4) and Mexico (IQR = 52.6–66.5). A similar, complementary picture was seen for the IA component (Table 1).

To estimate biases in ancestry representation among institutions, we explored differences in genetic ancestry estimates between institutions within countries (Supplementary Fig. S1). For most countries, no significant differences were observed except for Brazil (*P* = 0.003 for EUR, *P* < 0.001 for AFR, and *P* > 0.050 for IA and EAS components).

### Association between breast cancer subtypes and genetic ancestry

**Figure 2:**
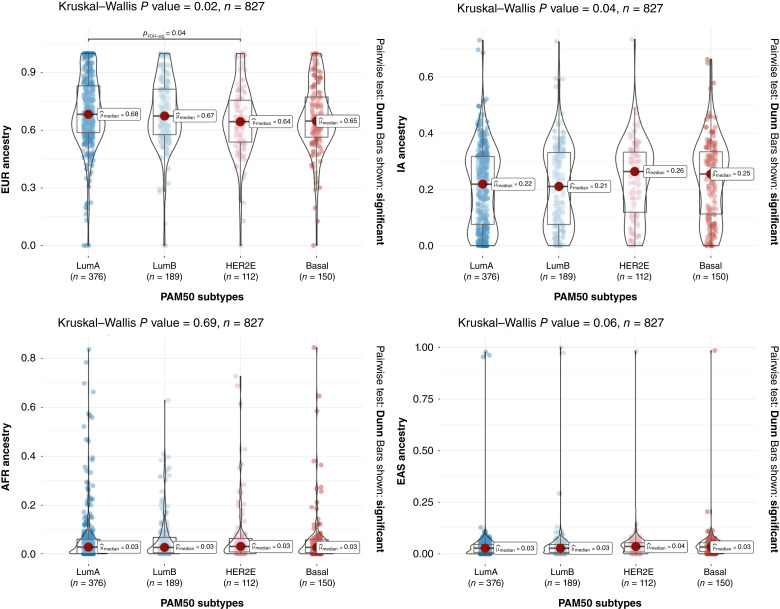
Distribution of PAM50 breast cancer subtypes according to the four most prevalent ancestral components in Latin America: European (EUR), Indigenous American (IA), African (AFR), and East Asian (EAS). Bars shown correspond to *post hoc* Dunn test pairwise comparisons <0.05. HER2E, HER2-enriched; LumA, luminal A; LumB, luminal B.

To study whether there was any association between ancestry and tumor subtypes, the median proportion of the different genetic ancestry components was compared among breast cancer subtypes defined both by IHC and PAM50. An analysis of differences of medians showed that EUR and IA proportions (but not AFR and EAS) were significantly different among PAM50 subtypes (Fig. 2). In the case of EUR ancestry, we could further demonstrate that the statistical signification was driven by the LumA–HER2E contrast and explained by the lower EUR ancestry associated with the HER2E subtype (LumA vs. HER2E adjusted *P* = 0.038 for EUR ancestry, Fig. 2). We could not see statistically significant differences in ancestry distribution for IHC-based subtypes (Supplementary Fig. S2).

Given the heterogeneity of the distribution of IA and AFR ancestries among countries, the limited number of patients, and the strong correlation between EUR and IA coefficients (−0.78, see Extended Fig. E3 in Supplementary Information), we first decided to perform subsequent analyses using the EUR ancestry proportion as a proxy of admixture. To evaluate whether there was an association between EUR ancestry and HER2 status, IHC or PAM50 subtypes in the context of a multivariable model, we conducted a multinomial logistic regression model with the subtype as the dependent variable and scaled EUR ancestry as the main predictor. The results from the univariate analysis showed that the HR− HER2+ subtype, but not HER2 status alone, was associated with lower EUR ancestry (Table 2, left). In the HR− HER2+ subtype, a 10% increase in EUR ancestry was significantly associated with an 11% decrease in the OR of presenting with this tumor subtype. The addition of the selected covariates kept the direction of the OR but affected the significance (Table 2). In the model with PAM50-intrinsic subtypes as the outcome, we also observed an inverse association between HER2E and EUR ancestry. In this case, a 10% increase in EUR ancestry was significantly associated with a 14% decrease in the odds of presenting HER2E breast cancer. The OR for the basal subtype was also significantly decreased (10%) with a 10% increase in EUR ancestry. The inverse association between the HER2E subtype and EUR ancestry maintained statistical significance with the addition of age, lymph node status, and AFR ancestry as covariates (Table 2). The incorporation of the variable country as an additional covariate did not significantly affect the OR but it did result in an increase in the *P* value of the model, rendering it statistically nonsignificant.

**Table 2:** Association between HER2 status, IHC and PAM50 subtypes, and EUR genetic ancestry (for every 10% increase in EUR or IA ancestry component)

			EUR ancestry		IA ancestry	
	Subtype	*n*	OR (CI)	*P* value	OR (CI)	*P* value
By HER status						
Univariate	HER2−	919^a^	Ref		Ref	
	HER2+		0.94 (0.86–1.03)	0.190	1.02 (0.91–1.14)	0.720
Nodal status	HER2−	903	Ref		Ref	
	HER2+		0.95 (0.87–1.04)	0.250	1.00 (0.89–1.12)	0.954
Nodal status + age at Dx	HER2−	903	Ref		Ref	
	HER2+		0.96 (0.87–1.05)	0.334	0.99 (0.88–1.12)	0.928
Nodal status + age at Dx + AFR ancestry	HER2−	903	Ref		Ref	
	HER2+		0.97 (0.88–1.07)	0.535	1.01 (0.90–1.14)	0.814
Nodal status + age at Dx + AFR ancestry + country	HER2−	903	Ref		Ref	
	HER2+		0.94 (0.84–1.06)	0.331	1.08 (0.91–1.27)	0.377
By IHC subtypes						
Univariate	HR+/HER2−	911 ^b^	Ref		Ref	
	HR+/HER2+		0.98 (0.88–1.10)	0.755	0.98 (0.85–1.13)	0.759
	HR−/HER2+		**0.89 (0.78–0.99)**	**0.040**	1.14 (0.97–1.34)	0.109
	HR−/HER2−		0.95 (0.86–1.05)	0.308	1.12 (0.99–1.27)	0.071
Nodal status	HR+/HER2−	895	Ref		Ref	
	HR+/HER2+		0.99 (0.88–1.12)	0.906	0.95 (0.82–1.11)	0.554
	HR−/HER2+		*0.88* (*0.78–1.00*)	*0.059*	1.11 (0.94–1.31)	0.205
	HR−/HER2−		0.97 (0.87–1.07)	0.556	1.08 (0.95–1.23)	0.226
Nodal status + age at Dx	HR+/HER2−	895	Ref		Ref	
	HR+/HER2+		1.01 (0.88–1.13)	0.844	0.94 (0.81–1.09)	0.406
	HR−/HER2+		0.87 (0.78–1.01)	0.065	1.11 (0.94–1.31)	0.217
	HR−/HER2−		0.98 (0.88–1.09)	0.716	1.07 (0.94–1.21)	0.307
Nodal status + age at Dx + AFR ancestry	HR+/HER2−	895	Ref		Ref	
	HR+/HER2+		0.99 (0.88–1.12)	0.906	0.95 (0.81–1.11)	0.550
	HR−/HER2+		*0.88* (*0.78–1.00*)	*0.059*	1.14 (0.96–1.36)	0.143
	HR−/HER2−		0.97 (0.87–1.07)	0.556	1.07 (0.94–1.23)	0.287
Nodal status + age at Dx + AFR ancestry + country	HR+/HER2−	895	Ref		Ref	
	HR+/HER2+		0.97 (0.84–1.13)	0.763	1.08 (0.87–1.34)	0.505
	HR−/HER2+		0.91 (0.77–1.06)	0.226	1.12 (0.89–1.41)	0.330
	HR−/HER2−		0.99 (0.87–1.14)	0.937	1.10 (0.92–1.31)	0.303
By PAM50						
Univariate	LumA	827 ^c^	Ref		Ref	
	LumB		0.97 (0.88–1.07)	0.572	1.01 (0.89–1.14)	0.848
	HER2E		**0.86 (0.77–0.97)**	**0.011**	**1.16 (1.00–1.35)**	**0.044**
	Basal		**0.90 (0.81–0.99)**	**0.047**	**1.17 (1.02–1.33)**	**0.021**
Nodal status	LumA	810				
	LumB		0.97 (0.88–1.08)	0.629	1.01 (0.89–1.15)	0.871
	HER2E		**0.86 (0.76–0.96)**	**0.009**	**1.16 (1.00–1.36)**	**0.050**
	Basal		0.92 (0.82–1.02)	0.120	1.13 (0.98–1.29)	0.095
Nodal status + age at Dx	LumA	810				
	LumB		0.97 (0.88–1.08)	0.631	1.01 (0.89–1.15)	0.875
	HER2E		**0.86 (0.76–0.97)**	**0.011**	*1.16* (*0.99–1.35*)	*0.056*
	Basal		0.93 (0.83–1.04)	0.182	1.11 (0.97–1.28)	0.130
Nodal status + age at Dx + AFR ancestry	LumA	810				
	LumB		0.97 (0.87–1.08)	0.635	1.01 (0.89–1.15)	0.847
	HER2E		**0.86 (0.76–0.98)**	**0.021**	**1.21 (1.03–1.42)**	**0.022**
	Basal		0.92 (0.81–1.03)	0.145	1.12 (0.97–1.30)	0.126
Nodal status + age at Dx + AFR ancestry + country	LumA	810				
	LumB		0.97 (0.85–1.10)	0.672	1.02 (0.85–1.22)	0.857
	HER2E		0.90 (0.78–1.04)	0.151	1.18 (0.95–1.45)	0.124
	Basal		0.96 (0.83–1.10)	0.547	1.07 (0.89–1.30)	0.461

Ancestry was modeled as a continuous variable and coefficients were scaled to reflect a 10% increase in ancestry. The number of individuals (*n*) in each analysis depends on the completeness of the variables used for adjustment. Bold numbers denote statistically significant differences, and italic numbers denote marginally nonsignificant values.

Abbreviations: CI, confidence interval; Dx, diagnosis; HER−, HER2 nonamplified; HER+, HER2 amplified; HER2E, HER2-enriched; LumA, luminal A; LumB, luminal B; OR: odds ratio.

a From the total of 951 genotyped patients, 32 had a HER2-missing status (Table 1), and from those, 16 lacked the nodal status.

b From the total of 951 genotyped patients, 40 had missing status of any of the HR or HER2 markers (Table 1), and from those, 16 lacked the nodal status.

c From the total of 951 genotyped patients, 124 had either missing data or belonged to the normal PAM50 subtype, which was not considered in this study (Table 1); from those, 17 also lacked the nodal status.

We further evaluated the association between the scaled IA ancestry and the breast cancer subtypes. This model did not reach significance for the IHC-based subtypes but showed a 16% increase in the odds of presenting HER2E breast cancer for every 10% additional IA ancestry (*P* = 0.044, Table 2, right). The incorporation of nodal status, age, and AFR ancestry covariables to the model rendered higher odds (21%) of presenting HER2E breast cancer for every 10% additional IA ancestry (*P* = 0.021). The inclusion of the “country” covariable abrogated statistical significance, although the direction and size of the OR was consistent with the association. In addition, in the univariate model, we could detect a 17% increase in the odds of presenting basal-like breast cancer for every 10% additional IA ancestry, but this association lost significance with the inclusion of covariables to the model (Table 2, right).

### Genetic ancestry, tumor subtype, and overall survival

We then evaluated the association between genetic ancestry and survival in univariate and adjusted Cox models, including the same covariates as in the previous analysis (age, lymph node status, AFR ancestry, and country) and adding the PAM50 subtypes (see Supplementary Information for a detailed description of the selection of covariables). Neither EUR nor IA ancestry was significantly associated with overall survival in univariate analysis (Table 3). Adjustment by confounders such as PAM50 subtype, age, AFR ancestry, and lymph node status resulted in an apparent increase in the hazard ratio with increasing EUR ancestry that was reverted by the addition of country as an additional confounder (Table 3).

**Table 3:** Univariate and multivariate Cox proportional hazard models for overall survival for every 10% increase of EUR or IA ancestry

	*n*	Hazard ratio (CI)	*P* value
EUR ancestry^a^			
Univariate	793	1.07 (0.97–1.18)	0.189
PAM50 subtypes	793	1.11 (1.00–1.23)	0.043
PAM50 subtypes + nodal status	780	1.14 (1.03–1.27)	0.014
PAM50 subtypes + nodal status + age at Dx	780	1.14 (1.02–1.27)	0.015
PAM50 subtypes + nodal status + age at Dx + AFR ancestry	780	1.15 (1.03–1.29)	0.012
PAM50 subtypes + nodal status + age at Dx +AFR ancestry + country	780	1.05 (0.92–1.21)	0.449
IA ancestry^a^			
Univariate	793	0.94 (0.83–1.06)	0.337
PAM50 subtypes	793	0.90 (0.79–1.02)	0.093
PAM50 subtypes + nodal status	780	0.86 (0.76–0.98)	0.021
PAM50 subtypes + nodal status + age at Dx	780	0.86 (0.75–0.98)	0.020
PAM50 subtypes + nodal status + age at Dx + AFR ancestry	780	0.85 (0.74–0.97)	0.013
PAM50 subtypes + nodal status + age at Dx + AFR ancestry + country	780	0.94 (0.79–1.11)	0.466

Abbreviations: CI, confidence interval; Dx, diagnosis.

a Ancestry was modeled as a continuous variable, and coefficients were scaled to reflect a 10% increase in the ancestry proportion. The number of individuals (*n*) in each analysis depends on the completeness of the variables used for adjustment.

## Discussion

There have been a limited number of studies conducted in diverse cohorts of Latin American patients with breast cancer that explored the association of genetic ancestry and tumor molecular characteristics (7, 8, 21, 22). These studies included women from Perú, Mexico, and Colombia. In this work, we further tested the association between genetic ancestry and breast cancer subtypes, defined both by IHC markers and PAM50, in the MPBCS cohort, which includes patients from Argentina, Brazil, Chile, Mexico, and Uruguay. The genetic ancestry distributions of these countries are heterogenous and close to those previously described (23–26), with our data showing that EUR ancestry is predominantly represented across all study sites in the different countries. In Chile and Mexico, the contribution of IA ancestry is higher compared with other countries. Additionally, Brazil shows an important proportion of AFR ancestry.

Our findings support previous observations of a higher frequency of HER2-dependent tumors in patients with increased IA and decreased EUR ancestry (8). In the MPBCS cohort, the association is seen only for HR− tumors, an observation already suggested by the Peruvian and Colombian studies (7). Moreover, even when the MPBCS cohort included both IHC and gene expression–based subtypes, the association seems to be more specific to the PAM50 HER2E subtype as the ORs were higher and the statistical significance was stronger for this intrinsic subtype than for its IHC counterpart. Interestingly, the HER2E subtype includes those tumors in which the HER2 pathway is active, regardless of the amplification status of the *ERBB2* gene. It is our hypothesis (to be explored) that the effect of IA ancestry on the HER2 pathway may not only be related to the amplification of *ERBB2* but to the activation of the HER2 pathway by various mechanisms.

Of note, the significance of the ORs was affected by the addition of the “country” variable, likely because of the power limitations when considering effects within each country. Sequentially adjusted models maintained the magnitude of the ORs and *P* values, suggesting that there are not strong mediators or confounders in the association between ancestry and subtype, except for the model including country.

In univariate Cox proportional hazard regression models, we showed that EUR and IA ancestries were not significantly associated with overall survival. However, adjustment for PAM50-intrinsic subtypes, age, AFR ancestry, and lymph node status rendered the model significant, showing an increase in mortality with higher EUR ancestry concomitant with a decrease in mortality with higher IA ancestry. The effect of the addition of country as a confounding variable in the model also abrogated these effects. Previous reports showed contradictory evidence of the effect of ancestry in breast cancer survival. On one hand, a lack of association between genetic ancestry and overall or cancer-specific survival was shown in a Californian Hispanic/Latina breast cancer cohort with homogeneous access to care (27). On the other hand, a more heterogeneous Hispanic/Latina cohort showed a twofold increase in mortality in women with more than 50% IA ancestry compared with women with 50% or less IA ancestry (28). Evidently, the complex and context-specific interplay between biological and nonbiological determinants of survival in admixed populations should be clarified with larger, comprehensive datasets from admixed cohorts (7).

This study has some limitations. First, the LACRN-MPBCS cohort is hospital-based and may not be representative of Latin American breast cancer in terms of clinical and/or pathologic characteristics. In addition, on average, participants from Mexico had lower IA ancestry than expected based on previous literature. Guadalajara and Sonora, known to have a more important Spanish contribution than other regions in Mexico (25, 29), was the source of patients in the Mexican LACRN-MPBCS cohort. It is also possible that patients recruited from Mexican sites had higher EUR ancestry than the general Mexican population. Alternatively, a higher proportion of EUR ancestry among LACRN-MPBCS participants could be explained by the previously described positive association between EUR genetic ancestry and breast cancer risk in Hispanic/Latina and Latin American women (10, 30–32). The skewed EUR ancestry proportion for the Mexican site and the limited proportion of IA ancestry in Brazil and Uruguay had an impact on the representation of the IA ancestry in the cohort, thus limiting the power of the analysis to evaluate the influence of IA in subtype distribution. Another limitation was the number of subjects for whom all data was available, which suggests that small but significant effects may have been missed in multivariable analyses because of missingness for some of the covariates.

In summary, the admixed LACRN-MPBCS cohort, with representation of Latin American countries that were not present in other studies, supports an association between IA ancestry and the HER2E breast cancer subtype. These results strengthen the hypotheses of the existence of either population-specific genetic variant(s) or of other ancestry-linked or correlated factors that affect HER2 expression in breast cancer in a consistent manner across different Latin American regions. We have already shown that SNPs specific to IA ancestry can affect cancer incidence in a subtype-specific manner (33–35). We can speculate that not yet discovered, ancestry-specific expression quantitative trait locus may be either affecting HER2 expression or signaling pathways relevant to HER2 expression (36). Other possible explanations may involve the existence of ancestry-specific splice variants (37) or genetic variants in other genes that affect the probability of HER2 pathway activation in tumor cells (38–40). On the other hand, nongenetic factors other than the ones included in our models may be acting as confounders on the association between genetic ancestry and the HER2E subtype (41, 42). This is especially relevant given that the association seen in this study is abrogated by the inclusion of “country” as a confounding variable. For example, Hispanic/Latino ancestry has been associated with lower socioeconomic status in the United States (9, 43). Individuals from lower socioeconomic backgrounds tend to seek medical attention at more advanced stages of breast cancer, often presenting with more aggressive tumor subtypes (44). This evidence may result from disparities in access to health services as a consequence of living in remote places and/or from a lack of awareness of slower-growing tumors (9, 45). These factors may induce a bias in the proportion of subtypes that reach medical care. We are actively pursuing studies that might shed light on the biological explanation for this observation.

### Ethics statement

The MPBCS was registered at ClinicalTrials.gov (identifier: NCT02326857) and adhered to the principles of the Declaration of Helsinki and local regulations. The study protocol was approved by the NCI Ethics Committee and local institutional review boards in each country. Before the study procedures, all participants signed study-specific written informed consent forms.

## Supplementary Material

Supplementary Figure S1 Distribution of the four ancestries (rows) according to the MPBCS participating institutions in each LACRN country (columns).

Supplementary Figure S2 Distribution of immunohistochemistry (IHC) breast cancer subtypes according to the four most prevalent ancestral components in Latin America.

Supplementary Information Supplementary information and List of LACRN Investigators
