# Supplementary material for: Genetic Ancestry, Intrinsic Tumor Subtypes, and Breast Cancer Survival in Latin American Women
Source: Cancer Res Commun. 2025 Jul 3;5(7):1070–81. doi: 10.1158/2767-9764.CRC-25-0014 (PMC12223717; doi:10.1158/2767-9764.CRC-25-0014)
Supplement: Supplementary Figure S1 — Distribution of the four ancestries (rows) according to the MPBCS participating institutions in each LACRN country (columns). [file crc-25-0014_supplementary_figure_s1_suppsf1.pdf]

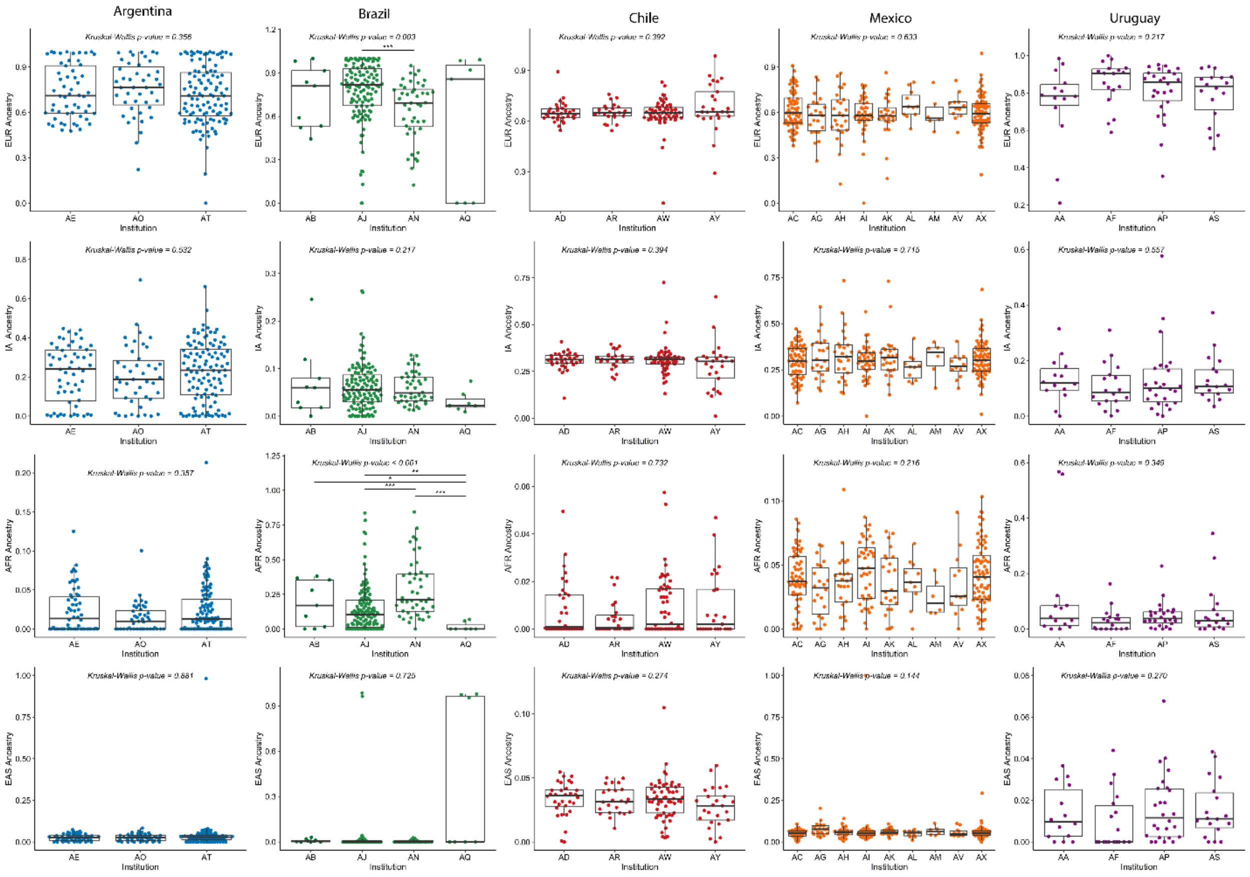

Supplementary Fig. S1: Distribution of the four ancestries (rows) according to the MPBCS participating institutions in each LACRN country (columns). EUR: European; IA: Indigenous 26
American; AFR: African; EAS: East Asian.
