# Supplementary material for: Genetic Ancestry, Intrinsic Tumor Subtypes, and Breast Cancer Survival in Latin American Women
Source: Cancer Res Commun. 2025 Jul 3;5(7):1070–81. doi: 10.1158/2767-9764.CRC-25-0014 (PMC12223717; doi:10.1158/2767-9764.CRC-25-0014)
Supplement: Supplementary Figure S2 — Distribution of immunohistochemistry (IHC) breast cancer subtypes according to the four most prevalent ancestral components in Latin America. [file crc-25-0014_supplementary_figure_s2_suppsf2.pdf]

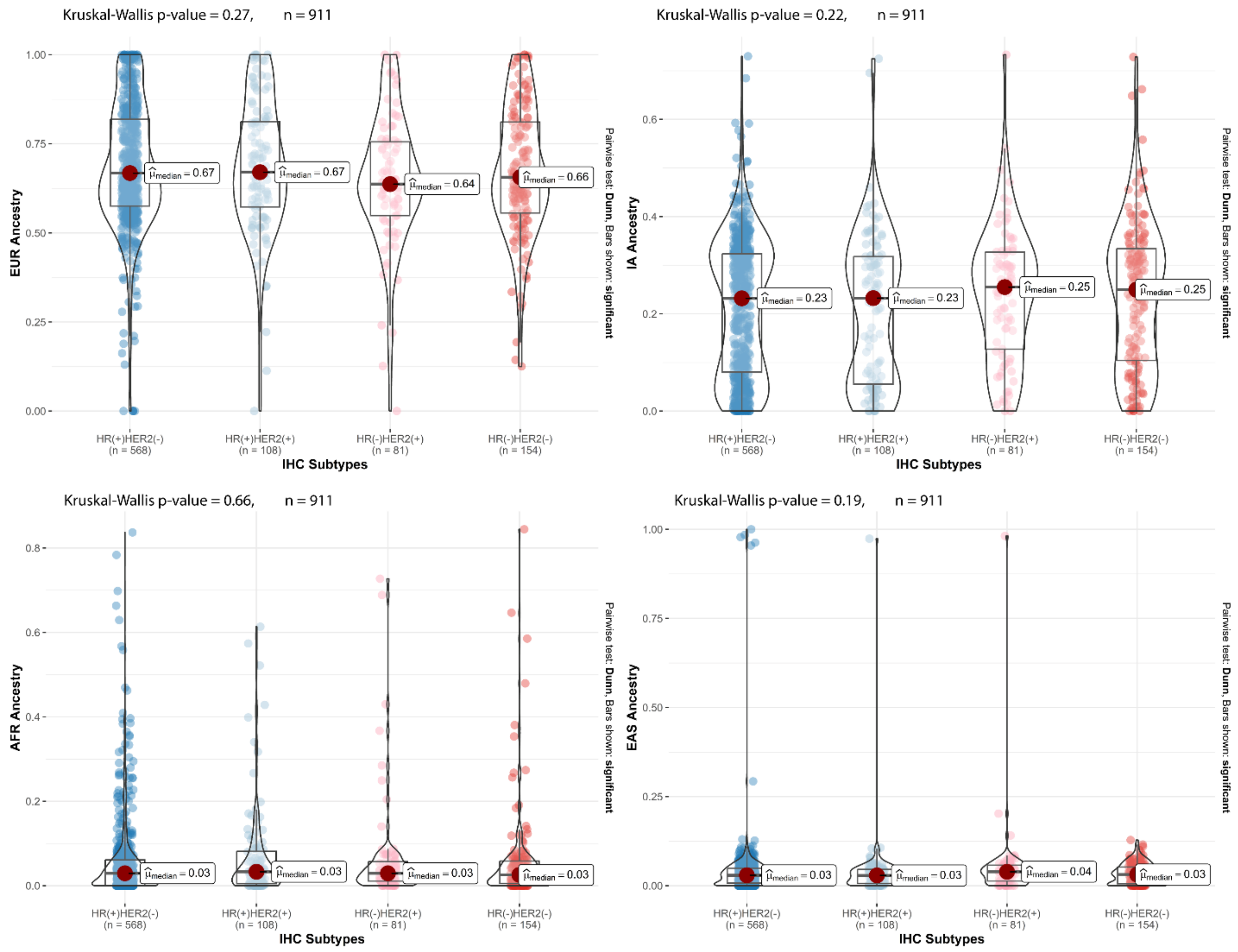

Supplementary Fig. S2: Distribution of immunohistochemistry (IHC) breast cancer subtypes according to the four most prevalent ancestral components in Latin America.

EUR: European; IA: Indigenous American; AFR: African; EAS: East Asian.

Bars shown correspond to post hoc Dunn's test pairwise comparisons <0.05.
