# Supplementary material for: Genetic Ancestry, Intrinsic Tumor Subtypes, and Breast Cancer Survival in Latin American Women
Source: Cancer Res Commun. 2025 Jul 3;5(7):1070–81. doi: 10.1158/2767-9764.CRC-25-0014 (PMC12223717; doi:10.1158/2767-9764.CRC-25-0014)
Supplement: Supplementary Information — and List of LACRN Investigators [file crc-25-0014_supplementary_information_suppsi.pdf]

Supplementary materials

Genetic ancestry, Intrinsic Tumor Subtypes, and Breast Cancer Survival in Latin American Women

Daniela Alves da Quinta, Dario Rocha, Cristian Yáñez, Renata Binato Gomes, Sheila Coelho Soares-Lima, Xiaosong Huang, Daiana Ganiewich, Valentina A. Zavala, Monica Sans, Alejandra Lopez-Vazquez, Jael Quintero, Olivia Valenzuela, Antonio Quintero-Ramos, Alicia del Toro-Arreola, Mauricio Cerda, Katherine Marcelain, Susanne Crocamo, Maria Aparecida Nagai, Dirce M Carraro, Marcia Maria Chiquitelli Marques, Jorge Gómez, Nora Artagaveytia, Adrián Daneri-Navarro, Bettina G Müller, Javier Retamales, Carlos Velazquez, Elmer A. Fernández, Osvaldo L. Podhajcer, the LACRN Investigators, Eliana Abdelhay, Ricardo A. Verdugo, Andrea S. Llera and Laura Fejerman.

List of materials

- Supplementary Information pp. 2-6
- List of LACRN Investigators pp. 7-15

Supplementary information

Association of epidemiological, clinical and pathological variables with European (EUR) ancestry in the whole cohort.

Differences in the distribution of EUR ancestry according to epidemiological, clinical and pathological variables usually correlated with breast cancer subtypes were tested in this subset of LACRN-MPBCS patients. For this analysis, Mann-Whitney's U test (i.e. Wilcoxon) (for variables with two categories) or Kruskal–Wallis tests (for three or more categories) were applied (stats, RRID:SCR_025968). For variables with three or more categories, pairwise comparisons were performed using post hoc Dunn's test with Benjamini-Hochberg p-value correction. For continuous variables such as age and BMI, association with EUR ancestry was tested by Spearman's rank correlation (stats, RRID:SCR_025968). We did not observe a correlation between BMI and EUR ancestry (Extended Fig. E1). Additionaly, differences in EUR ancestry proportion among histological grades or between ≤8 or >8 education years (as a proxy of lower vs higher socio-economic status) could not be proved with this dataset. On the contrary, age at diagnosis was positively correlated with EUR ancestry, although with a low Spearman's correlation coefficient (Extended Fig. E1). In addition, significatively lower EUR ancestry was found in locally advanced (stage IIB-III) compared to early (stage I-IIA) breast cancer patients ($p<0.001$); this effect was mostly pulled by the nodal status (negative vs. positive, $p<0.001$) with a marginal effect of clinical tumor stage (cT, $p=0.053$, Extended Fig. E1).

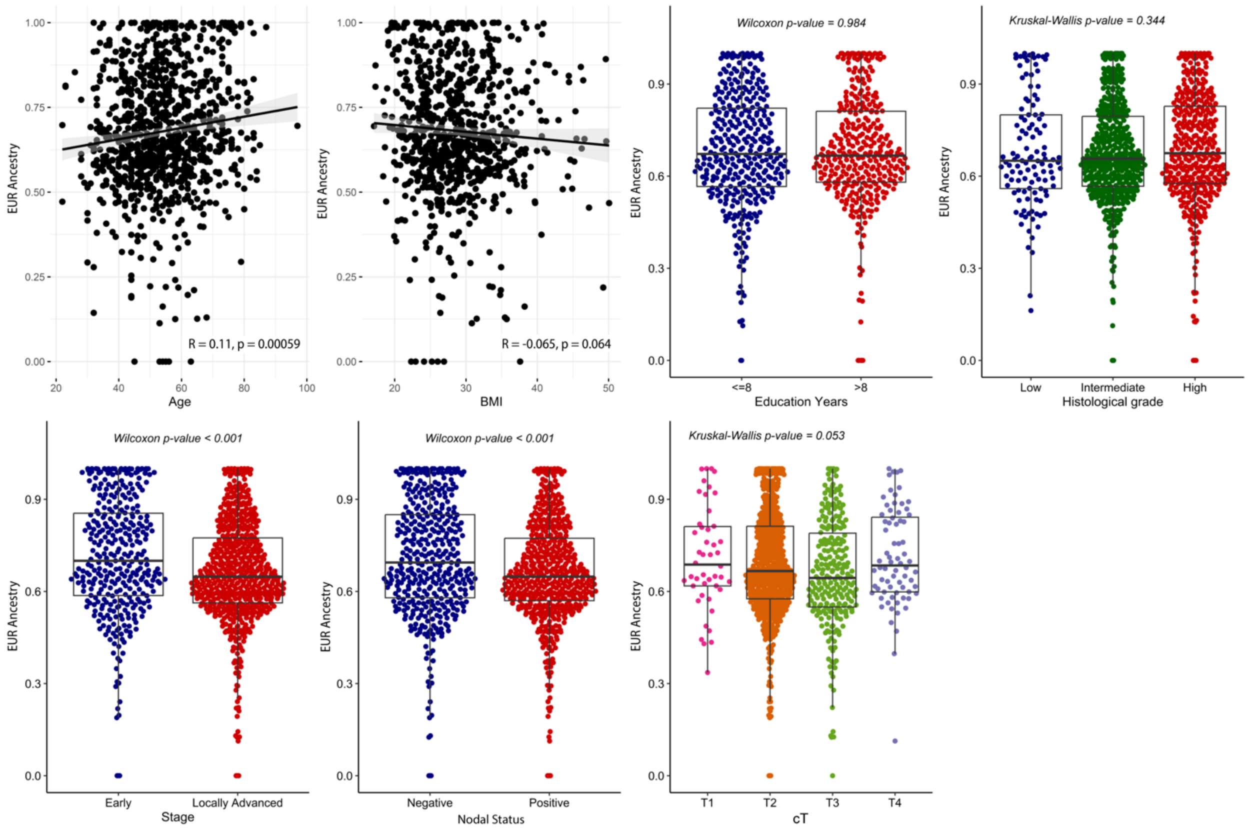

Extended Fig. E1: Distribution of demographic and pathological variables (age, BMI, education years, histological grade, stage, nodal status and cT) according to the European (EUR) ancestral component in MPBCS patients. BMI: body mass index; cT clinical tumor status.

Distribution of epidemiological, clinical, and pathological variables according to European (EUR) ancestry in each of the LACRN-MPBCS countries

The potential association between EUR ancestry and epidemiological, clinical and pathological variables was also tested by country (Extended Fig. E2). Most contrasts proved to be not significant in this subset of MPBCS patients, validating the whole-cohort approach for the study of the association between EUR ancestry and breast cancer subtype.

Argentina and Mexico showed a significant positive association between age and EUR ancestry (p=0.0310 and 0.0057, respectively). Argentina also showed a significant negative correlation between BMI and EUR ancestry (p=0.037) and Uruguay showed a higher proportion of EUR ancestry among node-negative cases (p=0.0071).

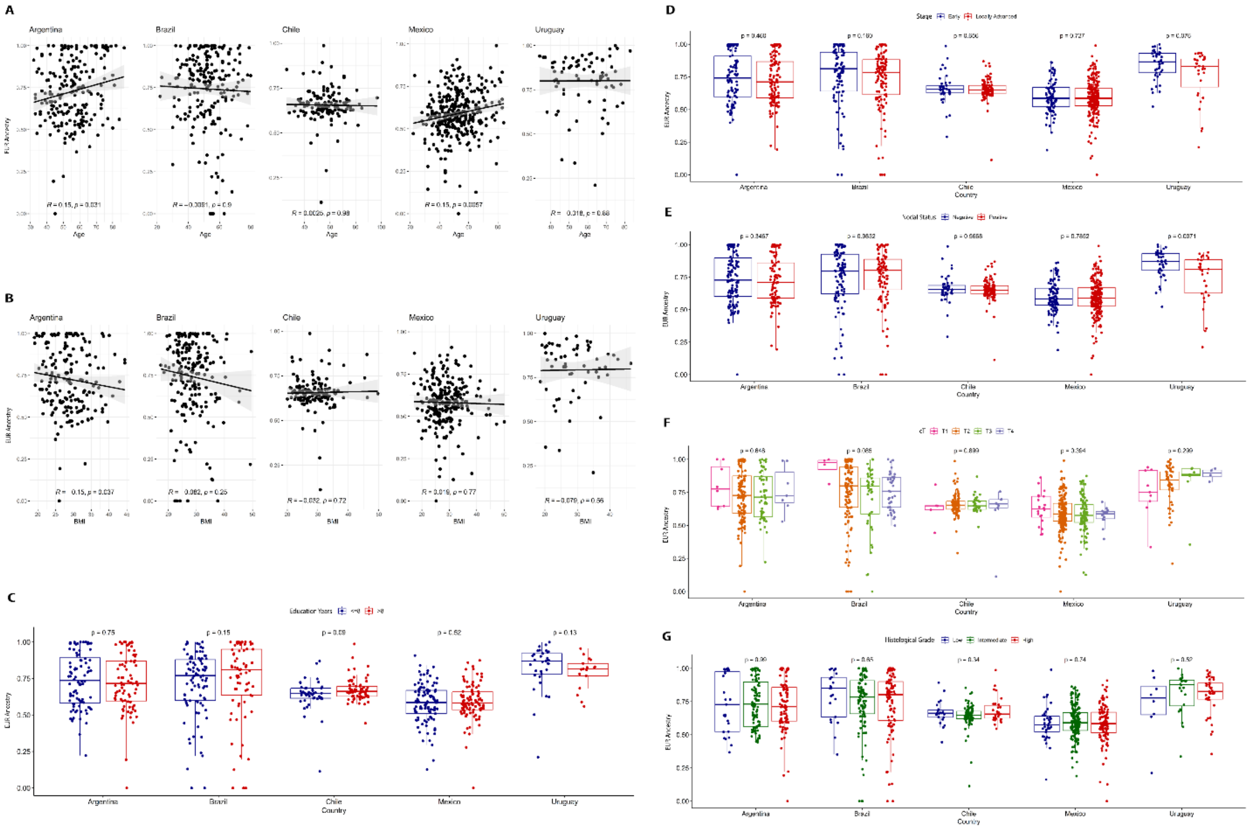

Extended Fig. E2: Distribution of epidemiological, clinical, and pathological variables (A. age; B. BMI; C. education years; D. stage; E. nodal status; F. cT; G. histological grade) according to European ancestry in each of the LACRN-MPBCS countries. BMI: body mass index; cT clinical tumor status.

Collinearity checks on potential confounders

To exclude highly correlated variables from model adjustment, we tested collinearity between clinical and demographic variables by pairwise comparisons using Spearman's rank correlation (for continuous variables), Cramer's V (for categorical variables), r coefficient (for continuous vs. 2 group-categorical variables; effect size from Wilcoxon test) or Eta-squared (for continuous vs. more than 2 group-categorical variables; effect size from Kruskal-Wallis test). Coefficients higher than 0.5 between two variables were considered as high.

As expected, EUR and IA ancestry were strongly inversely correlated (Extended Fig. E3). This evidence supported the use of EUR ancestry in our further analysis of association with subtype and survival. The AFR component, present in Brazil and Uruguay, was not highly correlated either to EUR or IA ancestry at the cohort level and represented a valid covariable for the model.

Clinical nodal and tumor status were strongly correlated with clinical stage (coefficients of 0.78 and 0.57, respectively), as they are parameters used to calculate stage. For this reason, we chose nodal status (i.e. negative vs positive) as the simplest and more complete confounder representative of stage.

Although country was a variable significantly associated to ancestry (see Table 1 in the manuscript), it was not substantially correlated with EUR ancestry, and did not surpass our threshold of 0.5 in the correlation with IA.

	Country	Age	BMI	Nodal Status	cT	Stage	Histological Grade	IA	EUR	AFR
Age	0.02									
BMI	-0.004	0.15								
Nodal Status	0.22	0.13	0.08							
cT	0.16	0.03	0.001	0.29						
Stage	0.20	0.14	0.05	0.78	0.57					
Histological Grade	0.11	0.01	-0.002	0.08	0.09	0.08				
IA	0.48	-0.09	0.05	0.15	0.01	0.14	0.01			
EUR	0.21	0.11	-0.06	0.12	0.005	0.13	0.0001	-0.78		
AFR	0.28	-0.11	0.03	0.03	0.01	0.05	0.001	-0.01	-0.35	
EAS	0.52	-0.08	0.03	0.12	0.01	0.13	0.005	0.79	-0.72	0.01

Stage and Nodal Status: 2-group categorical variable
Histological Grade and cT: more than 2-group categorical variable
Age, BMI, IA, EUR, AFR and EAS: continuous variables

r coefficient	: 2-group categorical vs. continuous (Wilcoxon)
Eta-squared	: >2-group categorical vs. continuous (Kruskal-Wallis)
Cramer's V	: categorical vs. categorical (Chi-squared)
Spearman's rank correlation	: continuous vs. continuous

Extended Fig. E3. Pairwise correlation between demographic and clinicopathological variables in MPBCS patients. BMI: body mass index; cT clinical tumor status; IA Indigenous American ancestry; EUR: European ancestry

From the results shown in previous sections, we decided to choose age, nodal status, AFR ancestry and country as the most relevant confounders in association analysis.

Effect of epidemiological, clinical, and pathological variables in overall survival

Log-rank analyses showed that breast cancer subtype ($p<0.001$ for both PAM50 and IHC subtypes), histological grade ($p=0.0013$), clinical stage ($p<0.001$), lymph node status ($p<0.001$) and clinical tumor status ($p<0.001$), but not age ($p=0.526$), BMI ($p=0.255$), AFR ancestry ($p=0.603$), country ($p=0.350$), or education years ($p=0.178$) were significantly associated with overall survival in this MPBCS subcohort. Age, AFR ancestry, and country are considered

standard variables associated with breast cancer survival and for this reason we decided to keep them in the model. Of note, we could not discard the possibility of a subtype-specific effect of the country in survival, as the statistical power was very low due to the relatively low number of patients of each subtype in the individual countries.

LIST OF LACRN INVESTIGATORS

Surname	Name	Suffix	Affiliation	City	Country
Abarca	Juan		Hospital Barros Luco Trudeau	Santiago	Chile
Abdelhay	Eliana	PhD	Instituto Nacional de Câncer	Rio de Janeiro	Brazil
Acevedo	Pamela		Hospital San José	Santiago	Chile
Acosta	Graciela		Hospital Municipal de Oncología María Curie	Buenos Aires	Argentina
Acosta	Gissel	MD	Hospital Universitario de Clinicas "Manuel Quintela"	Montevideo	Uruguay
Acosta	Ana	MSc	Universidad de Sonora	Hermosillo	México
Acosta Haab	Gabriela	MD	Hospital Municipal de Oncología María Curie	Buenos Aires	Argentina
Acosta-Torres	Keyla Teresa	BS	Hospital General de Zona No. 2, IMSS, Hermosillo	Hermosillo	México
Aghazarian	Marta	MD	Instituto Nacional del Cáncer	Montevideo	Uruguay
Aguayo	Carola	PhD	Instituto de Salud Pública	Santiago	Chile
Aizen	Bernardo	MD	Hospital Central de las Fuerzas Armadas	Montevideo	Uruguay
Alarcon-Lopez	Gustavo	MD	Hospital Integral de la Mujer en el Estado de Sonora	Hermosillo	México
Alcoba	Elsa	MD	Hospital Municipal de Oncología María Curie	Buenos Aires	Argentina
Almeida	Liz	MD,PhD	Instituto Nacional de Câncer	Rio de Janeiro	Brazil
Alonso	Isabel	MD	Centro Hospitalario Pereira Rossell	Montevideo	Uruguay
Alvarez	Ana	MD	Instituto de Oncología Angel Roffo	Buenos Aires	Argentina
Andrade	Viviane		Hospital de Câncer de Barretos	Barretos	Brazil
Angeles-Bueno	Wenceslao	MD	Hospital de Especialidades CMNO-IMSS	Guadalajara	Mexico
Arai	Roberto	PhD	Instituto de Câncer de São Paulo	São Paulo	Brazil
Arambula-Barreras	Priscila Elvira	MSc	Universidad de Sonora	Hermosillo	México
Arámburo-Rubio	Ma. Isabel	MD	Hospital General de Zona No. 2, IMSS, Hermosillo	Hermosillo	México
Araus	Estrellita		Hospital Barros Luco Trudeau	Santiago	Chile
Ardao	Gonzalo	MD	Hospital Central de las Fuerzas Armadas	Montevideo	Uruguay
Arellano-Jimenez	Lilia A	BS	Universidad de Guadalajara	Guadalajara	Mexico
Argandoña	Felipe		Hospital San Borja Arriarán	Santiago	Chile
Arias	Claudia	MD	Hospital Municipal de Oncología María Curie	Buenos Aires	Argentina
Armisen	Ricardo	MD,PhD	Universidad de Chile	Santiago	Chile
Artagaveytia	Nora	PhD	Hospital de Clínicas Manuel Quintela, Universidad de la República	Montevideo	Uruguay
Aspee	Mauricio	MD	Hospital Luis Tisne	Santiago	Chile
Assar	Rodrigo	PhD	Universidad de Chile	Santiago	Chile
Astiazarán-Rascón	Itzel Reneé	BS	Universidad de Sonora	Hermosillo	México
Astorga	Sebastian		Hospital San Borja Arriarán	Santiago	Chile
Avilés-Rodríguez	Maxwell	MD	Centro Estatal de Oncología	Hermosillo	México
Bailão Junior	Antônio	MD	Hospital de Câncer de Barretos	Barretos	Brazil
Barragan-Curiel	Adolfo E	MD	O.P.D. Nuevo Hospital Civil de Guadalajara	Guadalajara	Mexico
Barragan-Ruiz	Adelfo	MD	Hospital de Gineco-Obstetricia CMNO-IMSS	Guadalajara	Mexico
Bermudez	Fernanda		Programa Nacional para el Control del Cancer	Montevideo	Uruguay
Bernachin	Julia	MD	Hospital Universitario de Clinicas "Manuel Quintela"	Montevideo	Uruguay
Bernal-Herrera	Wilfrido	MD	Centro Estatal de Oncología	Hermosillo	México
Binato	Renata	PhD	Instituto Nacional de Câncer	Rio de Janeiro	Brazil
Bonet	Mara	MD	Instituto de Oncología Angel Roffo	Buenos Aires	Argentina
Bravo	Alicia I	MD	Hospital Regional de Agudos Eva Perón	Buenos Aires	Argentina
Brnich	Sarah	MD/PhD	Fundación Instituto Leloir-CONICET	Buenos Aires	Argentina

Bustamante	Claudio		Hospital San José	Santiago	Chile
Bustamante	Miguel Angel		Hospital San Borja Arriarán	Santiago	Chile
Bustos-Gomez	Julio	MD	O.P.D. Nuevo Hospital Civil de Guadalajara	Guadalajara	Mexico
Bustos-Rodriguez	Felipe de J	MD	O.P.D. Nuevo Hospital Civil de Guadalajara	Guadalajara	Mexico
Caballero-Jasso	Janett	MD	Hospital General de Zona No. 14, IMSS Hermosillo	Hermosillo	México
Calfuman	Angie		Hospital Luis Tisne	Santiago	Chile
Camejo	Natalia	MD	Hospital de Clínicas Manuel Quintela, Universidad de la República	Montevideo	Uruguay
Campos	Antonio Hugo José Froes Marques	MD,PhD	AC Camargo Cancer Center	São Paulo	Brazil
Campos	Mónica	MD	Hospital San Borja Arriarán	Santiago	Chile
Cano	Soledad		Instituto Nacional del Cáncer	Santiago	Chile
Canton-Romero	Juan C	MD	Hospital de Gineco-Obstetricia CMNO-IMSS	Guadalajara	Mexico
Cappetta	Ricardo	MD	Hospital Municipal Diego Thompson	Buenos Aires	Argentina
Carmona	Paulina	CRA	Grupo Oncológico Cooperativo Chileno de Investigación	Santiago	Chile
Carraro	Dirce Maria	PhD	AC Camargo Cancer Center	São Paulo	Brazil
Carrizo	Fernando	MD	Instituto de Oncología Angel Roffo	Buenos Aires	Argentina
Carvalho	André Lopes	MD,PhD	Hospital de Câncer de Barretos	Barretos	Brazil
Carvallo	Erika		Hospital San Borja Arriarán	Santiago	Chile
Carzoglio	Julio	MD	Instituto Nacional del Cáncer	Montevideo	Uruguay
Casalnuovo	Monica	MD	Hospital Municipal de Oncología María Curie	Buenos Aires	Argentina
Caserta	Benedicta	MD	Centro Hospitalario Pereira Rossell	Montevideo	Uruguay
Castillo	Alvaro		Hospital San José	Santiago	Chile
Castillo	César	MD	Hospital Barros Luco Trudeau	Santiago	Chile
Castro	Mónica	MD	Instituto de Oncología Angel Roffo	Buenos Aires	Argentina
Castro-Cervantes	Juan M	MD	Hospital de Especialidades CMNO-IMSS	Guadalajara	Mexico
Cataldi	Sandra	MD	Instituto Nacional de Cáncer	Montevideo	Uruguay
Cayota	Alfonso	PhD	Institut Pasteur de Montevideo	Montevideo	Uruguay
Cerda	Mauricio	PhD	Universidad de Chile	Santiago	Chile
Cerda	Yascara		Grupo Oncológico Cooperativo Chileno de Investigación	Santiago	Chile
Chammas	Roger	MD,PhD	Instituto de Câncer de São Paulo	São Paulo	Brazil
Chavez-Zamudio	Mario Alberto	MD	Hospital General Regional No. 1, IMSS, Obregon	Obregón	México
Chia	Loreto	MD	Hospital San José	Santiago	Chile
Chiarello	Elisa	MD	Hospital Municipal Diego Thompson	Buenos Aires	Argentina
Chirico	Daniela	BS	Fundación Instituto Leloir-CONICET	Buenos Aires	Argentina
Cisneros-Quirarter	Esther		Universidad de Guadalajara	Guadalajara	Mexico
Colombo	Alicia	PhD	Universidad de Chile	Santiago	Chile
Cordero-Bautista	Minor Raul	MD	Hospital General del Estado de Sonora	Hermosillo	México
Cornejo	Valeria	MD	Hospital San Borja Arriarán	Santiago	Chile
Corral-Villegas	Baldemar	MD	Centro Estatal de Oncología	Hermosillo	México
Cortés	Andrés	CRA	Grupo Oncológico Cooperativo Chileno de Investigación	Santiago	Chile
Cortés	Sandra	PhD	Pontificia Universidad Católica de Chile	Santiago	Chile
Cortes-Sanabria	Laura	MD/PhD	Hospital de Especialidades CMNO-IMSS	Guadalajara	Mexico
Cortez-Zamorano	German Salvador	BS	Universidad de Sonora	Hermosillo	México

Corvalan	Alejandro	MD	Grupo Oncológico Cooperativo Chileno de Investigación	Santiago	Chile
Crocamo	Susanne	MD/PhD	Instituto Nacional de Câncer	Rio de Janeiro	Brazil
Cruz	Adolfo	MD	Hospital Barros Luco Trudeau	Santiago	Chile
d'Aurora	Alba		Hospital San Borja Arriarán	Santiago	Chile
Daneri-Navarro	Adrian	MD/PhD	Universidad de Guadalajara	Guadalajara	Mexico
De la Fuente	Sandra	PhD	Universidad de Chile	Santiago	Chile
De la Peña	Soledad	MD	Centro Hospitalario Pereira Rossell	Montevideo	Uruguay
de Leon-Caballero	Roberto	MD	Hospital General del Estado de Sonora	Hermosillo	México
de Souza	Mirian	PhD	Instituto Nacional de Câncer	Rio de Janeiro	Brazil
Del Castillo	César	MD	Hospital San Borja Arriarán	Santiago	Chile
Del Toro-Arreola	Alicia	PhD	Universidad de Guadalajara	Guadalajara	Mexico
Del-Toro-Valero	Azucena	MD/PhD	Universidad de Guadalajara	Guadalajara	Mexico
Delgadillo-Cristerna	Raul	MD	Hospital de Especialidades CMNO-IMSS	Guadalajara	Mexico
Delgado	Lucía	MD	Hospital de Clínicas Manuel Quintela, Universidad de la República	Montevideo	Uruguay
Di Pretoro	Mirtha	PhD	Instituto de Oncología Angel Roffo	Buenos Aires	Argentina
Digonzelli	Andrea		Hospital Regional de Agudos Eva Perón	Buenos Aires	Argentina
Dreyer Breitenbach	Marisa	MD/PhD	Universidade Estadual do Rio de Janeiro	Rio de Janeiro	Brazil
El Ters	Jose	MD	Instituto Nacional del Cáncer	Montevideo	Uruguay
Escobar	Paula	MD	Hospital Luis Tisne	Santiago	Chile
Estolaza	Marcela		Hospital Luis Tisne	Santiago	Chile
Evangelista	Adriane Feijo	PhD	Hospital de Câncer de Barretos	Barretos	Brazil
Fanelli	Marcelo	MD	AC Camargo Cancer Center	São Paulo	Brazil
Farias	Paulo	MD,PhD	Instituto Nacional de Câncer	Rio de Janeiro	Brazil
Fernandez	Graciela	BS	Hospital Central de las Fuerzas Armadas	Montevideo	Uruguay
Fernández	Elmer	PhD	Universidad Católica de Córdoba, Centro de Investigaciones en Bioquímica Clínica e Inmunología-CONICET	Córdoba	Argentina
Fernández	Jorge	PhD	Instituto de Salud Pública	Santiago	Chile
Fernández	Wanda	MD	Hospital San Borja Arriarán	Santiago	Chile
Filgueiras	Natalia	MD	Hospital Municipal de Oncología María Curie	Buenos Aires	Argentina
Flaks	Diego	MD	Hospital Municipal de Oncología María Curie	Buenos Aires	Argentina
Flores-Ayala	Edgar G	MD	Instituto Jalisciense de Cancerologia	Guadalajara	Mexico
Flores-Marquez	Maria R	MD	Hospital de Especialidades CMNO-IMSS	Guadalajara	Mexico
Franco-Hughes	David	BS	Universidad de Sonora	Hermosillo	México
Franco-Topete	Ramon A	MD/PhD	O.P.D. Hospital Civil de Guadalajara, Universidad de Guadalajara	Guadalajara	Mexico
Franco-Topete	Karina	BS	O.P.D. Nuevo Hospital Civil de Guadalajara	Guadalajara	Mexico
Freire	Jimena	BS	Instituto Nacional del Cáncer	Montevideo	Uruguay
Fresno	Cristobal	PhD	Universidad Católica de Córdoba	Córdoba	Argentina
Gabay	Carolina	MD	Instituto de Oncología Angel Roffo	Buenos Aires	Argentina
Gabrielli	Romina	BS	Centro Hospitalario Pereira Rossell	Montevideo	Uruguay
Gaete	Fancy	MD	Hospital Luis Tisne	Santiago	Chile
Gallegos	Mario		Hospital San Borja Arriarán	Santiago	Chile
Gamboa	Jorge	MD	Hospital San Borja Arriarán	Santiago	Chile
Ganiewich	Daiana	Bioeng	Fundación Instituto Leloir-CONICET	Buenos Aires	Argentina

Garbovesky	Carlos	MD	Hospital Municipal de Oncología María Curie	Buenos Aires	Argentina
Garcia-Gaeta	Ricardo	MD	Universidad de Guadalajara	Guadalajara	Mexico
Garcia-Martinez	Alma C	MSc	Universidad de Guadalajara	Guadalajara	Mexico
García-Munguía	Rubén Alejandro	MD	Hospital de Ginecopediatría, IMSS, Hermosillo	Hermosillo	México
Garibay-Escobar	Adriana	PhD	Universidad de Sonora	Hermosillo	México
Gimenez	Liliana	MD	Instituto de Oncología Angel Roffo	Buenos Aires	Argentina
Gómez Silveira	Hector	MD	Hospital Municipal Diego Thompson	Buenos Aires	Argentina
Gomez-Del Toro	Mariana M	MD	Universidad de Guadalajara	Guadalajara	Mexico
Gonzalez	Marcela	BSc	Hospital Regional de Agudos Eva Perón	Buenos Aires	Argentina
Gonzalez	Alicia	BS	Hospital Universitario de Clinicas "Manuel Quintela"	Montevideo	Uruguay
González	Germán	BS	Universidad Católica de Córdoba	Córdoba	Argentina
González-Mondaca	César Osbaldo	MD	Hospital General de Zona No. 2, IMSS, Hermosillo	Hermosillo	México
Gonzalez-Ramirez	Leivy P	PhD	Universidad de Guadalajara	Guadalajara	Mexico
Gonzalez-Ulloa	Beatriz	MD	Hospital de Especialidades CMNO-IMSS	Guadalajara	Mexico
Gorostidy	Susana	MD	Instituto de Oncología Angel Roffo	Buenos Aires	Argentina
Grass	Mariela		Instituto Nacional del Cáncer	Montevideo	Uruguay
Greif	Gonzalo	PhD	Institut Pasteur de Montevideo	Montevideo	Uruguay
Guerrero	Marisol	MD	Hospital San José	Santiago	Chile
Guevara-Torres	Alfonso G.	MD	Centro Estatal de Oncología	Hermosillo	México
Gutierrez	Lorena		Hospital San Borja Arriarán	Santiago	Chile
Gutierrez-Rubio	Susan A	PhD	Universidad de Guadalajara	Guadalajara	Mexico
Hannois	Adrián	MD	Hospital Regional de Agudos Eva Perón	Buenos Aires	Argentina
Hart	Andrew	PhD	Universidad de Chile	Santiago	Chile
Härtel	Steffen	PhD	Universidad de Chile	Santiago	Chile
Henriquez	Marcos	MD	Hospital Barros Luco Trudeau	Santiago	Chile
Hernandez-Franco	Miriam E	MSc	Universidad de Guadalajara	Guadalajara	Mexico
Hernandez-Guevara	Rafael	MD	Hospital General Regional No. 1, IMSS, Obregon	Obregón	México
Herrera-Miramontes	Manuel I	MD	Universidad de Guadalajara	Guadalajara	Mexico
Horton	Graciela	MD	Hospital Municipal de Oncología María Curie	Buenos Aires	Argentina
Ibañez	Gladys	MD	Hospital San José	Santiago	Chile
Ipiña	Martín	MD	Instituto de Oncología Angel Roffo	Buenos Aires	Argentina
Jalfin	Beatriz	BSc	Hospital Regional de Agudos Eva Perón	Buenos Aires	Argentina
Jara	Lilian	PhD	Universidad de Chile	Santiago	Chile
Jara	Raul		Hospital Luis Tisne	Santiago	Chile
Jaramillo	Maria Luisa		Hospital San Borja Arriarán	Santiago	Chile
Jimenez	Maria Eugenia		Hospital Barros Luco Trudeau	Santiago	Chile
Jimenez-Moreno	Victor M	MD	Hospital de Gineco-Obstetricia CMNO-IMSS	Guadalajara	Mexico
Ju	Hugo	CRA	Grupo Oncológico Cooperativo Chileno de Investigación	Santiago	Chile
Juárez Rusjan	Nazareth	BSc	Instituto de Oncología Angel Roffo	Buenos Aires	Argentina
Juneman	Karen	MD	Hospital Luis Tisne	Santiago	Chile
Kerr	Ligia Maria	MD,PhD	Hospital de Câncer de Barretos	Barretos	Brazil
Krupelis	Alejandra		Hospital Municipal de Oncología María Curie	Buenos Aires	Argentina
Larios-Jimenez	Flor Esmeralda	PhD	Universidad de Guadalajara	Guadalajara	Mexico
Latorre	Jose Domingo	MD	Hospital Municipal Diego Thompson	Buenos Aires	Argentina
Laviña	Guillermo	MD	Hospital Universitario de Clinicas "Manuel Quintela"	Montevideo	Uruguay
Lavista	Fernando	MD	Hospital Central de las Fuerzas Armadas	Montevideo	Uruguay

León-Duarte	Irma Leticia	MD	Hospital General del Estado de Sonora	Hermosillo	México
Lescano	Alberto	MD	Hospital Municipal de Oncología María Curie	Buenos Aires	Argentina
Lezano	Verónica	CRA	Grupo Oncológico Cooperativo Chileno de Investigación	Santiago	Chile
Llera	Andrea S	PhD	Fundación Instituto Leloir-CONICET	Buenos Aires	Argentina
Lopez	Rossana Mendoza	PhD	Instituto de Câncer de São Paulo	São Paulo	Brazil
López-Cervantes	Jose Guillermo	MD	Universidad de Sonora	Hermosillo	México
Lopez-Muñoz	Miguel Enrique	MEng	Universidad de Sonora	Hermosillo	México
Lopez-Vazquez	Alejandra	MSc	Universidad de Sonora	Hermosillo	México
Loria	Dora	PhD	Instituto de Oncología Angel Roffo	Buenos Aires	Argentina
Luque	Alejandra	MD	Hospital Central de las Fuerzas Armadas	Montevideo	Uruguay
Maass	Alejandro	PhD	Universidad de Chile	Santiago	Chile
Maciel	Maria do Socorro	MD	AC Camargo Cancer Center	São Paulo	Brazil
Maldonado	Silvina	MD	Hospital Regional de Agudos Eva Perón	Buenos Aires	Argentina
Mangone	Flavia Rotea	PhD	Instituto de Câncer de São Paulo	São Paulo	Brazil
Mansilla	Jorge		Universidad de Chile	Santiago	Chile
Marcelain	Katherine	PhD	Universidad de Chile	Santiago	Chile
Mariani	Carolina	CRA	Grupo Oncológico Cooperativo Chileno de Investigación	Santiago	Chile
Marques	Marcia Maria Chiquit	PhD	Hospital de Câncer de Barretos	Barretos	Brazil
Martinez-Arriaga	Reyna J	PhD	Universidad de Guadalajara	Guadalajara	Mexico
Martinez-Ramirez	Hector R	MD/PhD	Hospital de Especialidades CMNO-IMSS	Guadalajara	Mexico
Martins	Marcela	MD	Instituto de Câncer de São Paulo	São Paulo	Brazil
Maya-Gonzalez	Alma G		Universidad de Guadalajara	Guadalajara	Mexico
Mazzaferri	Brenda	MD	Hospital Municipal Diego Thompson	Buenos Aires	Argentina
Menini	Mariana	MD	Hospital Central de las Fuerzas Armadas	Montevideo	Uruguay
Míguez	Silvia		Hospital Municipal de Oncología María Curie	Buenos Aires	Argentina
Milans	Soledad	MD	Hospital Universitario de Clinicas "Manuel Quintela", Instituto Nacional del Cancer	Montevideo	Uruguay
Montes	Soledad		Instituto Nacional del Cáncer	Santiago	Chile
Morales-Hernández	Ana Verónica	BS	Universidad de Sonora	Hermosillo	México
Moran-Mendoza	Andres de J	MD	Hospital de Gineco-Obstetricia CMNO-IMSS	Guadalajara	Mexico
Morga-Villela	Giberto	MD	Hospital de Especialidades CMNO-IMSS	Guadalajara	Mexico
Morong	Carla	MD	Hospital San Borja Arriarán	Santiago	Chile
Müller	Bettina	MD	Instituto Nacional del Cáncer	Santiago	Chile
Muñoz	Homero	Comp Eng	Programa Nacional para el Control del Cancer	Montevideo	Uruguay
Muse	Ignacio Miguel	MD	Programa Nacional para el Control del Cancer	Montevideo	Uruguay
Mussetti	Carina	MD	Centro Hospitalario Pereira Rossell	Montevideo	Uruguay
Mussetti	Eduardo	MD	Centro Hospitalario Pereira Rossell	Montevideo	Uruguay
Nagai	Maria Aparecida	PhD	Instituto de Câncer de São Paulo	São Paulo	Brazil
Najar-Acosta	Luis J	BS	Universidad de Guadalajara	Guadalajara	Mexico
Napolitano e Ferreira	Elisa	PhD	AC Camargo Cancer Center	São Paulo	Brazil
Navarro-Ruiz	Nancy E	BSN	Universidad de Guadalajara	Guadalajara	Mexico
Noblía	Cristina	MD	Instituto de Oncología Angel Roffo	Buenos Aires	Argentina
Nunes	João Soares	MD,PhD	Hospital de Câncer de Barretos	Barretos	Brazil
Núñez	Daniela	MD	Hospital Regional de Agudos Eva Perón	Buenos Aires	Argentina
Núñez	Fabiola		Hospital Luis Tisne	Santiago	Chile
Oceguera-Villanueva	Antonio	MD	Instituto Jalisciense de Cancerologia	Guadalajara	Mexico
Onari	Nilton	MD	Hospital de Câncer de Barretos	Barretos	Brazil
Oropeza-De Anda	Emma M	BSP	Universidad de Guadalajara	Guadalajara	Mexico
Ortega-Tirado	David	MSc	Universidad de Sonora	Hermosillo	México

Ortiz-Martinez	Miguel Angel	MD	Hospital General Regional No. 1, IMSS, Obregon	Obregón	México
Osório	Cynthia Aparecida Bueno de Toledo	MD,PhD	AC Camargo Cancer Center	São Paulo	Brazil
Paiva	Carlos Eduardo	MD	Hospital de Câncer de Barretos	Barretos	Brazil
Peñaloza	Paulina	MD	Hospital Luis Tisne	Santiago	Chile
Peredo-Navarro	Miguel	BS	Hospital de Especialidades CMNO-IMSS	Guadalajara	Mexico
Pereira	David	MD	Instituto de Oncología Angel Roffo	Buenos Aires	Argentina
Perez-Michel	Laura	MD	Hospital General Regional No. 1, IMSS, Obregon	Obregón	México
Pino	Francisca		Hospital Barros Luco Trudeau	Santiago	Chile
Pino	Tania		Hospital San José	Santiago	Chile
Pinto	Natalia		Hospital Luis Tisne	Santiago	Chile
Pizarro	Jessica		Hospital Barros Luco Trudeau	Santiago	Chile
Podhajcer	Osvaldo L	PhD	Fundación Instituto Leloir-CONICET	Buenos Aires	Argentina
Pressa	Carlos	MD	Hospital Universitario de Clinicas "Manuel Quintela"	Montevideo	Uruguay
Quintero	Jael	PhD	Universidad de Sonora	Hermosillo	México
Quintero-Ramos	Antonio	PhD	Universidad de Guadalajara	Guadalajara	Mexico
Ramirez	Enrique		Hospital Municipal de Oncología María Curie	Buenos Aires	Argentina
Ramirez-Rosales	Gladys E	MD	Universidad de Guadalajara	Guadalajara	Mexico
Ramis	Claudia	MD	Hospital San José	Santiago	Chile
Ramos-Ramirez	Maritza	MD	Universidad de Guadalajara	Guadalajara	Mexico
Rascon-Alcantar	Adela	MD	Hospital Infantil del Estado de Sonora	Hermosillo	México
Ravaglio	Silvana	BS	Hospital Central de las Fuerzas Armadas	Montevideo	Uruguay
Reis	Rui M	PhD	Hospital de Câncer de Barretos	Barretos	Brazil
Retamales	Javier	MD	Grupo Oncológico Cooperativo Chileno de Investigación	Santiago	Chile
Richard	Francois		Grupo Oncológico Cooperativo Chileno de Investigación	Santiago	Chile
Rios-Méndez	Omar	BS	Hospital General del Estado de Sonora	Hermosillo	México
Rivera-Claisse	Ernesto	MD	Centro Estatal de Oncología	Hermosillo	México
Robles-Zepeda	Ramón E.	PhD	Universidad de Sonora	Hermosillo	México
Rocha	Iara Santana	MD	Hospital de Câncer de Barretos	Barretos	Brazil
Rodriguez	Natalia	MD	Hospital Barros Luco Trudeau	Santiago	Chile
Rodriguez	Vilma	MD	Hospital Barros Luco Trudeau	Santiago	Chile
Rodriguez	Maria Teresa		Hospital San José	Santiago	Chile
Rodriguez	Robinson	MD	Hospital Central de las Fuerzas Armadas	Montevideo	Uruguay
Rodriguez-Gonzalez	Diego	MD	Universidad de Guadalajara	Guadalajara	Mexico
Roela	Rosemeire A	PhD	Instituto de Câncer de São Paulo	São Paulo	Brazil
Romero-Gomez	Ana M	MD	Universidad de Guadalajara	Guadalajara	Mexico
Rosales	Cristina	MD	Hospital Municipal de Oncología María Curie	Buenos Aires	Argentina
Rosales-Sandoval	Ana M	BSN	Hospital de Especialidades CMNO-IMSS	Guadalajara	Mexico
Rubio-Chavez	Lidia A	BS	Universidad de Guadalajara	Guadalajara	Mexico
Rubio-Plascencia	Omar V	BSP	Universidad de Guadalajara	Guadalajara	Mexico
Russo	Florencia	Tecn.	Hospital Regional de Agudos Eva Perón	Buenos Aires	Argentina
Sabini	Gaciela	MD	Programa Nacional para el Control del Cancer	Montevideo	Uruguay
Saffie	Isabel	MD	Hospital Luis Tisne	Santiago	Chile
Salas-Gonzalez	Efrain	MD	Hospital de Gineco-Obstetricia CMNO-IMSS	Guadalajara	Mexico
Samaniego	Brenda	MSc	Universidad de Sonora	Hermosillo	México

San Martino	Julio	MD	Hospital Municipal Diego Thompson	Buenos Aires	Argentina
Sanchez-Llamas	Benito	MD	Hospital de Gineco-Obstetricia CMNO-IMSS	Guadalajara	Mexico
Sanchotena	Verónica	MD	Hospital Municipal de Oncología María Curie	Buenos Aires	Argentina
Sat-Muñoz	Daniel	MD	Hospital de Gineco-Obstetricia CMNO-IMSS	Guadalajara	Mexico
Savignano	Mariana	MD	Instituto de Oncología Angel Roffo	Buenos Aires	Argentina
Scapulatempo Neto	Cristovam	MD,PhD	Hospital de Câncer de Barretos	Barretos	Brazil
Segovia	Laura	MD	Hospital Barros Luco Trudeau	Santiago	Chile
Sendoya	Juan M	PhD	Fundación Instituto Leloir-CONICET	Buenos Aires	Argentina
Senna	Max Mano	MD,PhD	Instituto de Câncer de São Paulo	São Paulo	Brazil
Silva	Carolina		Hospital San Borja Arriarán	Santiago	Chile
Silva-Garcia	Aida A	MD	O.P.D. Hospital Civil de Guadalajara, Universidad de Guadalajara	Guadalajara	Mexico
Silvera	Jaime	MD	Centro Hospitalario Pereira Rossell	Montevideo	Uruguay
Small	Isabele	MsC	Instituto Nacional de Câncer	Rio de Janeiro	Brazil
Soares	Fernando	MD,PhD	AC Camargo Cancer Center	São Paulo	Brazil
Soares	Iberê	MD,PhD	Instituto de Câncer de São Paulo	São Paulo	Brazil
Soares dos Santos	Silvana		AC Camargo Cancer Center	São Paulo	Brazil
Sobrosa de Mello	Evandro	MD,PhD	Instituto de Câncer de São Paulo	São Paulo	Brazil
Sola	José Antonio	MD	Instituto Nacional del Cáncer	Santiago	Chile
Sorín	Irene	MD	Hospital Regional de Agudos Eva Perón	Buenos Aires	Argentina
Sosa	Anabella	Secretary	Hospital Regional de Agudos Eva Perón	Buenos Aires	Argentina
Sosa	Alejandra	MD	Programa Nacional para el Control del Cancer	Montevideo	Uruguay
Sosa	Claudio	MD	Centro Hospitalario Pereira Rossell	Montevideo	Uruguay
Soto	Sandra		Instituto Nacional del Cancer	Montevideo	Uruguay
Souza	Cristiano de Pádua	MD,PhD	Hospital de Câncer de Barretos	Barretos	Brazil
Spangenberg	Lucía	PhD	Institut Pasteur de Montevideo	Montevideo	Uruguay
Steffanof	Gustavo	PhD	Instituto Nacional de Câncer	Rio de Janeiro	Brazil
Straminsky	Florencia	BS	Fundación Instituto Leloir-CONICET	Buenos Aires	Argentina
Tapia	Mónica		Hospital Luis Tisne	Santiago	Chile
Tapia-Llanos	Raziel O	BS	Universidad de Guadalajara	Guadalajara	Mexico
Tavares-Macias	Geronimo M	MD	Hospital de Especialidades CMNO-IMSS	Guadalajara	Mexico
Temperley	Guillermo	MD	Hospital Municipal de Oncología María Curie	Buenos Aires	Argentina
Terzieff	Veronica	MD	Centro Hospitalario Pereira Rossell	Montevideo	Uruguay
Teti	Vicente	MD	Hospital Municipal de Oncología María Curie	Buenos Aires	Argentina
Tognarelli	Javier	PhD	Instituto de Salud Pública	Santiago	Chile
Toledo	Verónica	MD	Hospital Luis Tisne	Santiago	Chile
Toro	Paulina		Hospital Luis Tisne	Santiago	Chile
Torres	Roberto	MD	Instituto Nacional del Cáncer	Santiago	Chile
Torres-Palomares	Mariana		Universidad de Guadalajara	Guadalajara	Mexico
Trinchero	Alejandra	BSc	Hospital Regional de Agudos Eva Perón	Buenos Aires	Argentina
Troyo-San Roman	Rogelio	Bioeng	Universidad de Guadalajara	Guadalajara	Mexico
Urbano	Hernan		Hospital Barros Luco Trudeau	Santiago	Chile
Vacca	Nicolas	Comp. Eng.	Programa Nacional para el Control del Cancer	Montevideo	Uruguay
Vaimberg	Daniel	MD	Hospital Regional de Agudos Eva Perón	Buenos Aires	Argentina
Valencia-Peña	María Lourdes	MSc	Universidad de Sonora	Hermosillo	México

Valenzuela	Olivia	PhD	Universidad de Sonora	Hermosillo	México
Vaselevich	Maria Lujan	MD	Hospital Municipal Diego Thompson	Buenos Aires	Argentina
Vazquez-Nares	Jaime	MD	Instituto Jalisciense de Cancerología	Guadalajara	Mexico
Velazquez	Carlos	PhD	Universidad de Sonora	Hermosillo	México
Velez-Gomez	Ezequiel	MD	O.P.D. Nuevo Hospital Civil de Guadalajara	Guadalajara	Mexico
Venegas-Godinez	Laura N	MD	Universidad de Guadalajara	Guadalajara	Mexico
Vercelli	Patricia	MD	Hospital Regional de Agudos Eva Perón	Buenos Aires	Argentina
Verdugo	Ricardo	PhD	Universidad de Chile	Santiago	Chile
Vieira	René Aloisio da Costa	MD,PhD	Hospital de Câncer de Barretos	Barretos	Brazil
Vilensky	Marta	BSc	Instituto de Oncología Angel Roffo	Buenos Aires	Argentina
Villarubias	María José	MD	Hospital Regional de Agudos Eva Perón	Buenos Aires	Argentina
Villegas-Gómez	Manuel Isaac	MSc	Universidad de Sonora	Hermosillo	México
Viña	Stella	BSc	Instituto de Oncología Angel Roffo	Buenos Aires	Argentina
Vornetti	Silvia	MD	Hospital Municipal de Oncología María Curie	Buenos Aires	Argentina
Watanabe	Anapaula Hidemi Uema	MD	Hospital de Câncer de Barretos	Barretos	Brazil
Zagame	Livia	MD	Instituto Jalisciense de Cancerologia	Guadalajara	Mexico
Zamorano	Carlos		Hospital Barros Luco Trudeau	Santiago	Chile
Zapata	Luis	MD	Hospital Barros Luco Trudeau	Santiago	Chile
Zlatar	Zdenka	CRA	Grupo Oncológico Cooperativo Chileno de Investigación	Santiago	Chile
